# A Qualitative Exploration of Consumers’ Perceived Impacts, Behavioural Reactions, and Future Reflections of the EU Tobacco Products Directive (2017) as Applied to Electronic Cigarettes

**DOI:** 10.1177/1179173X20925458

**Published:** 2020-06-19

**Authors:** Emma Ward, Claudia Anholt, Sarah Gentry, Lynne Dawkins, Richard Holland, Caitlin Notley

**Affiliations:** 1 Norwich Medical School, University of East Anglia, Norwich, UK; 2 Centre for Addictive Behaviours Research, School of Applied Sciences, London South Bank University, London, UK; 3 Leicester Medical School, University of Leicester, Leicester, UK

**Keywords:** Electronic cigarettes, vaping, ENDS, tobacco regulation, tobacco policy, Tobacco Products Directive, qualitative research, consumer views, nicotine

## Abstract

**Background:**

Electronic cigarette regulations included in the Tobacco Products Directive (TPD), Article 20, implemented in Europe by May 2017, aimed to improve safety for e-cigarette consumers, and prevent uptake among non-smokers, particularly young people. Before implementation, there were significant concerns from consumers, industry, and some in the scientific community about the potential negative impact of the TPD on people using e-cigarettes to remain abstinent from smoking. To date, there is limited evidence on how the TPD has affected consumers. This study aimed to add insight into how consumers perceived and experienced the regulations.

**Methods:**

Qualitative data, collected between March 2018 and March 2019, relating to participant views of the TPD were extracted from 160 interviews/extended surveys of e-cigarette consumers as part of a wider study into e-cigarette use trajectories (ECtra study). Data were thematically analysed.

**Results:**

Awareness of the TPD among consumers was not universal. Participants’ smoking behaviour did not appear to be influenced by the legislation. Participants were reassured by manufacturing regulations and requirements for ingredients labels. Participants responded negatively to changes perceived to cause inconvenience and extra plastic waste. The product restrictions prompted some participants to purchase non-compliant products illegally, potentially putting their safety at risk.

**Conclusions:**

E-cigarette regulation should focus on ensuring product safety. Raising awareness of the TPD among consumers and smokers could be beneficial.

## Introduction

E-cigarettes are now the most popular smoking cessation aid chosen by UK smokers^1^ and have been shown to be an effective aid for smoking cessation.^2,3^ However, although they are recognised as being much less harmful than tobacco smoking,^4^ the long-term health effects are not yet known^5^ and there is concern, particularly in the United States, about the potential for youth uptake, and subsequent nicotine addiction among never smokers.^6^ A number of studies have demonstrated that e-cigarette liquid and aerosol does contain potentially harmful compounds (eg, carbonyl compounds with carcinogenic potential, heavy metals, respiratory irritants^7,8^; the levels of which can vary depending on the nature of the device and usage conditions).^9-11^ Although these levels are typically far lower than those found in tobacco smoke,^7,11^ the effects of repeated inhalation on health are yet to be quantified. Encouragingly, studies of respiratory health have demonstrated fewer respiratory symptoms in exclusive e-cigarette users (vapers) compared with smokers^12^ and smokers with asthma or chronic obstructive pulmonary disease (COPD) report symptom improvement when switching to e-cigarettes.^13,14^ Similarly, studies measuring urinary biomarkers of exposure to cancer, cardiovascular and respiratory disease typically record far lower levels of these markers in e-cigarette users compared with smokers.^15-19^ Nevertheless, the absolute risk of long-term e-cigarette use (vaping) is yet to be determined, and there have been reported outbreaks of adverse reactions related to the misuse of e-cigarettes (eg, by vaping adulterated or unregulated e-liquids).^20^ Ensuring that e-cigarette devices and e-liquids are as safe as they can be, falls partly under the remit of legislation and regulation.

Regulation of e-cigarettes varies considerably around the world, from no legalisation in around half of countries to a complete ban on sales in 29 countries.^21^ In the European Union (EU), the revised Tobacco Products Directive (TPD) was implemented between May 2016 and May 2017. The TPD included e-cigarettes (Article 20), introducing regulations including refill liquid containers limited to a maximum volume of 10 ml with no higher than 20 mg/ml nicotine concentration; refillable tanks and cartridges (the reservoir included in the e-cigarette which holds the e-liquid) were not to exceed a capacity of 2 ml; all vaping products to include a health warning label stating that ‘this product contains nicotine which is a highly addictive substance’, and liquid packaging to list ingredients. In addition, the TPD prohibits specific hazardous ingredients and requires producers to notify their country’s relevant regulatory authority before launching a product to market.^22^

The TPD regulations were introduced with the intention to increase e-cigarette safety by setting minimum standards and providing information to consumers to allow them to make informed choices, while also protecting children and deterring never-smokers from trying e-cigarettes by limiting marketing and including nicotine warning labels. The vaping community and some scientists raised concerns that a reduction in nicotine strength might stop smokers from quitting using e-cigarettes, or cause users to relapse to smoking, as the lower strength may not satisfy cravings.^23-25^ However, cross-sectional survey evidence suggests that the limits on nicotine strength do not appear to have influenced consumers previously using non-compliant high strengths to be more susceptible to returning to smoking, as feared.^26^ UK policy makers were worried that price increases would drive consumers to the black market, potentially putting their health at risk.^27^ It is not clear to what extent this concern is warranted, but consumers and retailers have found legal methods to overcome the restrictions resulting from the TPD. For example, some consumers stockpiled high nicotine concentrations of e-liquid for use in mixing their own liquids,^28^ and many retailers embraced product innovation, such as selling ‘nicotine shots’.^29^ These are TPD compliant 10 ml bottles containing unflavoured nicotine of the maximum legally permitted strength of 20 mg/ml; users dilute the nicotine in larger bottles of 0 mg/ml liquid, enabling them to legally possess a large bottle of e-liquid tailored to their desired nicotine strength.

Evidence investigating how the TPD is experienced by consumers is limited, but is vital for policy makers to consider when developing e-cigarette policy. Indeed, the UK Government has recently made a commitment to review the TPD restrictions relating to nicotine strength limits, tank restrictions, advertising, and ingredient notifications, in light of the United Kingdom’s exit from the EU.^30^ This is the first study to our knowledge using in-depth qualitative exploration of consumers’ perceptions and experiences of the TPD regulation since its implementation.

## Methods

The data drawn upon to answer the research question ‘How do vapers perceive and experience the EU Tobacco Products Directive (2017) as applied to e-cigarettes (Article 20)?’ are taken from Phase 2 of a wider longitudinal study, the ‘E-Cigarettes Trajectories Study’ (ECtra), exploring patterns of e-cigarette use in relation to preventing smoking relapse through longitudinal mixed methods data collection.^31,32^ The study received ethical approval from the UEA Faculty of Medicine and Health Sciences Research Ethics Committee (project reference: 2017/2018 – 106).

### Recruitment and sampling

Between March 2018 and March 2019, 184 participants took part in Phase 2 of the study, 12 to 18 months after they initially participated in Phase 1 of the study (2016-2017). The eligibility criteria included people aged 18 years or above, who had attempted to use an e-cigarette for smoking cessation. Participants were originally recruited into Phase 1 of the study through word of mouth, local press articles, university bulletins, vape shops, and social media. The ECtra study was initially designed to be an interview study, but due to over recruitment, the research team devised an alternative survey version of the interview which was shared with project enquirers who were unable to participate in an interview, and on social media.

Forty interview participants were recruited for Phase 1 and 37 participants were interviewed for Phase 2 (one participant declined Phase 2 participation due to personal reasons and 2 did not respond to contact attempts). With regard to the survey, 371 participants were recruited for Phase 1 and 147 participated in Phase 2 (77 did not provide an email address at Phase 1, 57 started the Phase 2 survey but did not complete, and 90 did not respond to emailed requests to complete the Phase 2 survey). At Phase 2, only participants who identified themselves as being resident in the EU were asked about the TPD; this included all interview participants and 125/147 survey participants. Two EU-based survey participants did not provide an answer to the TPD question resulting in a final sample of n = 160.

### Procedure

The Phase 2 online survey and interview topic guide were developed in consultation with lay consultants. Both data collection tools asked similar questions. The questions were derived from findings illuminated from Phase 1 of the ECtra study,^29,31,33^ and related to relapse pathways^34^ and partnership working between healthcare professionals and the vaping industry.^35^ In addition, a question about the perceived impact of the TPD was included to explore experiences of the legislation which had come into full effect in May 2017, just after Phase 1 data collection had completed. Only data generated from that one question were analysed for this paper. Both data collection instruments included the same question phrasing (Appendix 1).

### Analysis

Participants gave informed consent before taking part in a confidential online survey or telephone (25)/face-to-face (12) interview. Interviews were recorded, transcribed verbatim, and anonymised. Surveys were administered via the Qualtrics online survey platform^36^ using a hyperlink and data were downloaded once the survey closed. Participant responses to the TPD question were extracted from interview transcripts and downloaded survey data, then were uploaded to NVivo 12 qualitative analysis software.^37^ The extracts were coded using a standardised thematic analysis method^38^: C.A. coded data for latent and semantic content within the interview sample data, with E.W. coding a subset of approximately 10% of interview data to check for coding consistency. Codes were iteratively reviewed and sorted into subthemes and overarching themes by C.A. in discussion with E.W. until data analysis saturation was achieved with no new codes being generated. This coding structure was applied deductively to coding of the survey sample by C.A. and E.W., allowing for inductive coding and iterative sorting of themes as needed. Following this, the themes were written up with illustrative quotes for each identified theme by C.A. and E.W. As is typical in qualitative research, this sometimes resulted in the recoding and categorising of the data. The final analytical write up was critically reviewed by EW resulting in a comprehensive interpretation of the data in relation to the research question and the final thematic structure agreed by C.A., E.W., and C.N.

## Results

Profile of participant characteristics is reported in Table 1. Just over a quarter of all participants were female (41, 27.3%), ages ranged from 22 to 79 years (mean = 49, SD = 12.37), three participants were from Black, Asian, and minority ethnicities (BAME), and 45.9% (67) were employed in managerial, professional, or technical occupations.^39^ Most (133, 89.9%) participants identified as being resident in the United Kingdom due to this being where the UK based research team recruited, and that English language was used. The vast majority of participants were vaping and abstinent from tobacco (139, 86.9%), 10 participants had relapsed (4 dual using both tobacco and vaping), and 11 were no longer using either e-cigarettes or tobacco. Of those using e-cigarettes at the time of Phase 2 data collection, the median length of use was 4 years (range: 1-9) and the vast majority did not plan to quit using e-cigarettes (124, 86.7%). The survey and interview samples differed mainly on gender and vaping status. No participants reported regular use of smokeless ‘heat-not-burn’ tobacco products.

**Table 1.** Profile of participant characteristics (n = 160).

	Interview sample, n = 37	Survey sample,^a^ n = 123	Combined int/survey sample, n = 160
Gender			
Male	51.4% (19)	79.6% (90)	72.7% (109)
Female	48.6% (18)	20.4% (23)	27.3% (41)
Age (n = 150)			
Range (years)	49: 22-71	53: 26-79	57: 22-79
Mean (years)	42 (SD: 14.32)	52 (SD: 10.71)	49 (SD: 12.37)
Ethnicity			
White	100% (37)	97.3% (108)	98% (145)
BAME		2.7% (3)	2% (3)
Managerial, professional, or technical occupation	37.8% (14)	48.6% (53)	45.9% (67)
Resident location			
United Kingdom	97.3% (36)	87.4% (97)	89.9% (133)
Other EU	2.7% (1)	12.6 (14)	10.1% (15)
T2 vaping status			
Vaping and abstinent from tobacco	62.2% (23)	97.3% (116)	86.9% (139)
Abstinent from both vaping and tobacco	16.2% (6)	4.1% (5)	6.9% (11)
Relapsed to tobacco (dual using)	5.4% (2)	1.6% (2)	2.5% (4)
Relapsed to tobacco (not vaping)	16.2% (6)		3.8% (6)
Approx. years using e-cig at T2 (for vaping participants only, n = 79)			
Range	8: 1-8	9: 1-10	9: 1-9
Median	4	4	4
T2 future intentions to continue/discontinue vaping (for vaping participants only, n = 143)			
No plans to quit vaping	96% (24)	87.7% (100)	86.7% (124)
Plans to quit vaping	4% (1)	15.3% (18)	13.3% (19)

Abbreviations: BAME, Black, Asian, and minority ethnicities; EU, European Union; SD, standard deviation; TPD, Tobacco Products Directive.

a Only included participants identifying as resident in EU who answered the TPD question.

Overarching themes relating to participants’ perceptions of the TPD regulation were identified (Table 2) and are discussed in turn using illustrative quotes from both survey and interview data. Numbers of participants discussing each individual theme are not provided in line with common qualitative practices,^40^ as the aim of the analysis was to identify possible perspectives and experiences of the TPD, rather than infer prevalence of experience. Forty-six (28.7%) participants reported no awareness of the TPD or any personal behavioural reactions/ negative impacts. In these instances, participants’ opinions about the TPD regulations were elicited. Within the sample, males were four times more likely to report a behavioural reaction or negative impact compared to women, χ^2^(2, N = 150) =13.04, *P* < .01, odds ratio (OR) = 3.93.

**Table 2.** Summary of themes identified and example quotations relating to participants’ perceptions and experiences of the Tobacco Products Directive regulatory changes.

Theme	Example quotation
Perceived Impacts – how consumers perceive the TPD has affected them	
Low awareness and no perceived impact	‘It has not affected me’. (Survey No. 48)
Reassurance about e-liquid ingredients – makes vaping feel safer	‘I think the TPD has been good at cleaning up the market from foreign imports which could contain poor ingredients’. (Survey No. 42)
Limits consumer choice–makes vaping more expensive, less accessibility to effective products	‘Strength restrictions mean many early stage vapers fail because they can’t get the nicotine level needed and those who do stay on are using more eliquid than would otherwise be needed. This means more vapour is produced leading to more complaints’. (Survey No. 60)
Inconveniences consumer – makes vaping more complicated through increased refilling and bottle purchasing/carrying	‘Tank Capacity (2 ml) – To me this seems pointless and unnecessary’. (Survey No. 22)
Increases plastic waste	‘People have to fill up tanks more often, carry extra bottles of juice and the 10ml bottles greatly increased plastic waste’. (Survey No. 26)
Behavioural Reactions – how consumers perceive they have responded to the changes	
Already using compliant products – no reaction	‘The e-liquid I’ve been buying has always only come in 10 mils so I guess [the TPD] hasn’t really crossed my consciousness at all’. (InterviewNo.18)
Stocking up on non-compliant products pre-TPD–including large quantities of nicotine	‘I home mix and stocked up on 72mg before the deadline’. (SurveyNo.101)
Market reactions such as nicotine shots	‘10 mil bottles are ridiculous, suppliers get round this by doing nicotine shots. This needs to change fast’. (SurveyNo.25)
Buying from black market/abroad– concerns about safety	‘I have actively defied the TPD, importing directly from China’. (SurveyNo.33)
Future Reflections – what consumers believe regulation should focus on	
To avoid confusing switchers, removal of nicotine warning labels on vaping products not containing nicotine should be considered	‘Nicotine labels = ridiculous, laughable idiocy’. (Survey No. 69)
To reduce possible health risks, further regulation of e-liquid ingredients/product safety desired	‘Perhaps there’s potential for further regulation because obviously there’s a myriad of people selling vaporising products now’. (Interview No. 1)

Abbreviation: TPD, Tobacco Products Directive.

### Perceived impacts

Irrespective of whether or not participants were aware of the legislation, most participants supported some form of regulation designed to promote consumer safety:

You do need regulation in things like this, because it is going into somebody’s body [. . .] The fact that it’s got batteries in it, you definitely need regulation. (Interview No. 31)

The most popular change initiated by the TPD was the requirement for ingredients lists on e-liquid bottles. This reassured most participants to some extent, allowing them to know what they were inhaling and to exert choice over which ingredients they were consuming:

You would expect to find the ingredients on food that you buy, wouldn’t you? So why shouldn’t they be on the vaping liquids? So yeah, I think they should have them, and I suppose as well, that can help you avoid certain things. (Interview No. 30)

Some participants were using compliant products prior to the TPD implementation and, therefore, did not feel that their purchasing was negatively restricted:

Not worried about strength as I use a fairly low strength anyway. (Survey No. 126)

Although a small number of participants had noticed a decrease in price due to competition between shops, many participants had noted an increase in price:

The legislation has made it difficult for me to cost-effectively buy consumables for vaping (coils, e-liquid). In a large number of cases, hardware has also increased in price. (Survey No. 102)

The restriction on e-liquid strength to 20 mg/ml was considered too low by many participants. Although no participants mentioned lapsing themselves as a result of the changes, several commented that it may prevent smokers from converting to vaping as they needed over 20 mg/ml of nicotine when they initially stopped smoking:

20mg is useless for heavy smokers wanting to switch, I needed 36mg to start 5 years ago, so more will fail and go back to smoking. (Survey No. 41)

Some participants commented that products they normally used were no longer sold as they did not comply with the new regulations, resulting in having to buy new parts which, in addition to increasing the cost, was also inconvenient:

It was more annoying, basically I had to buy a new tank because the coils didn’t fit in the same tank anymore, or like stuff was discontinued. (Interview No. 27)

One participant, however, did comment that, although inconvenient, the restrictions on tanks had improved functionality and safety:

The pro is that they don’t leak, and that is a real plus and no vaper wants a leaky tank and they never bothered before about making sure they were all leak proof, so that’s a good thing. (Interview No. 31)

The reduction in tank size to 2 ml and refill bottles to 10 ml was an unpopular change among the participants who reported having to refill the tank more frequently and carry several smaller refill bottles around with them. In addition, they reported that it made the vaping process more difficult as everything is now smaller:

TPD restrictions are well meaning but misguided. It is completely pointless to restrict the size of bottles and tanks, in fact it may even add to the problem. If you need to constantly keep topping up a small tank, you need to carry bottles of liquid, and restricting bottle size does not make people carry less liquid. (Survey No. 49)

Some participants commented that reduced tank size could discourage smokers to switch due to the added inconvenience:

Convenience is a big factor in helping smokers transition to vaping so I think it’s a big shame. Specifically: It’s a [pain] to keep filling up your tank every few hours. (Survey No. 88)

In addition to finding the smaller bottles inconvenient, many participants were uncomfortable with the extra plastic waste that was being generated as a result of using far more plastic bottles of e-liquid than before the legislation:

I’m more concerned about the environmental impact of many, many more tiny plastic bottles being produced. This is a backward step for the environment. (Survey No. 78)

### Behavioural reactions

Many participants did not discuss any behavioural reactions, because they were not aware of the TPD and/or they were using compliant products pre-regulation. Reported reactions included participants who had pre-empted the TPD and began home mixing e-liquid enabling them to create higher nicotine strength liquid and/or keep the price of e-liquid low rather than buying more expensive 10 ml bottles. They had bought the nicotine base before the legislation came into force:

I stocked up on high strength nicotine solution (72 mg/ml) before the TPD came into force – I have about 4 years supply left. (Survey No. 24)

Participants reported benefitting from retailers stocking nicotine shots which could be added to larger bottles of 0 mg e-liquids:

The bottle size doesn’t stop people purchasing the same amount of e-juice, and of course there are ways (legal ways) around the legislation which suppliers provide, such as shake and mix type purchasing (purchasing nicotine shots to add to a larger flavour bottle). (Survey No. 2)

Many participants had bought non-compliant products online from the black market via countries where the regulations did not apply:

If you order it from outside the UK, they will send out the bigger tank glasses without a second thought. (Interview No. 36)

Since I continue to use 24mg/ml, which is prohibited under the TPD, I have no alternative but to source products from the black market. (Survey No. 136)

China, United States, and the Isle of Man were the most commonly mentioned places participants bought non-compliant products. These participants believed that this was the only way they could purchase higher strength nicotine and larger tanks that contributed to the vaping set up which worked well for them. A couple acknowledged the impact on domestic business and commented that they would have preferred to support local shops if the products were available:

Small businesses in the UK are throttled and for little need. (Survey No. 139)

Some also had concerns that purchasing in this way put their safety at further risk because they did not trust the quality of black market foreign products:

Making my own liquid is more of a problem. Getting nicotine concentrate isn’t as easy, sourcing it nowadays means getting what could be dodgy stuff [. . .] I still have some nicotine base in the freezer. When that runs out, I’ll have a problem, but I’ll just have to use the black market and risk getting ‘bad’ ingredients. (Survey No. 111)

### Future reflections

Many vapers, although pleased with the ingredients listings/restrictions as outlined above, wanted further regulation on the content of liquids and safety of devices. They felt that this would give them much needed reassurance that the products they were using were as safe as possible:

I think that’s a good thing [. . .] It’s alluding to a degree of quality control, you know, the actual chemicals that they do put in the liquid, but I think they could have probably gone a bit further and you know just made that any chemicals or flavouring, they know are safe or not known to be carcinogenic. (Interview No. 2)

Some participants who wanted further regulation for product safety were not aware that e-liquid ingredients had already been regulated as part of the TPD legislation:

I’d be happy to see more regulations. I don’t know how much has been done in this area already, on the liquids, on the additives and the alcohols and the sort of diacetyl and things like this, the extra things that could be harmful in it. I mean I know there is a lot of choice out there now [. . .] I don’t know how strict they are in what chemicals go into the product [. . .] And I actually don’t know if that is being regulated enough yet. (Interview No. 8)

In contrast to inclusion of ingredients lists, the vast majority of participants thought that the inclusion of the ‘contains nicotine’ warning label, including on hardware and 0 mg liquids packaging, was nonsensical and confusing:

Mandatory warning labels on mods or empty atomizers saying they contain nicotine are, in my opinion, plain ridiculous and serve no purpose. (Survey No. 114)

I think it’s misinformation in terms of all the kit have got to have it marked ‘this product contains nicotine’ which as we well know a lot of them don’t. (Interview No. 6)

Many participants felt that the warning label may deter people switching from smoking, although no participant reported that the warning label made them think twice about vaping. A couple proposed that warning labels should instead focus on communicating reduced harm messages to smokers on tobacco cigarette packets in an attempt to nudge them into switching to less harmful vaping:

[Tobacco packaging] is all standardized in terms of the colour and you know big health warnings on there and pictures of you know people with their throats falling out and stuff! [. . .] I think if they, rather than it all just being you know pictures of, all the horrible things they put on there[. . .] is to maybe actually have some information about alternatives like vaping, you know, vaping is 95% safer. I think those kind of nudging ways of encouraging people, you know as well as the health warnings would be helpful. (Interview No. 2)

## Discussion

To the best of our knowledge, this is the first study of consumers’ perceptions and reported experiences of the EU-TPD. Mixed reported experiences of TPD were illuminated, ranging from no impact or awareness, to illegal purchasing of non-compliant products. Aspects of the TPD that participants agreed with, irrespective of whether or not they were previously aware of the regulations, were greater manufacturing regulations and full ingredients labelling. Participants wanted reassurance about the safety of the products they were using and would welcome further regulations addressing this, mirroring the UK Government’s commitment to fund research into product toxicity.^30^ A previous UK survey of smokers, ex-smokers, and vapers, showed that awareness for most of the TPD regulations was less than 10%.^26^ It can be inferred that, like many participants in this study, the vast majority of e-cigarette users are not familiar with the legislation. It is reassuring that this participant group did not knowingly experience any negative impacts. However, these participants perceived vaping products to be currently unregulated and, in some cases, wanted regulations that were already in place. It may be helpful to raise awareness of the TPD among consumers, as smokers may be put off switching if they think products are not subject to any regulation or control.

It is encouraging that no participants commented that they had relapsed as a direct result of the restrictions, as most had adapted to the changes or were already using compliant products, but some participants had decided to purchase illegal products, such as nicotine strength over 20 mg/ml. For some participants, the nicotine strength liquid they originally used to quit smoking was no longer available. However, most tobacco quitters using e-cigarettes today may not need to use illegal strengths, because technology has advanced alongside the TPD implementation, meaning that devices are more powerful and effective at delivering nicotine and that lower strength e-liquids can be satisfying.^41^ It is worth noting though that many new users lack vaping experience and may initially use less sophisticated devices which may require higher strength liquid to be effective or satisfying.^42,43^ In addition, using advanced devices with high power alongside lower nicotine results in compensatory puffing causing vapers to use more e-liquid which can increase exposure to potential toxicants and carcinogens.^9,10^ The nicotine strength limit, however, has been suggested as a possible reason for the UK having comparatively lower rates of youth vaping compared to North America,^44^ which, if evidenced, should be carefully considered when reviewing the legislation. Stricter marketing restrictions have also been suggested as a possible reason for comparatively lower rates of youth vaping. Interestingly, advertising was not discussed by any of the participants, indicating established e-cigarette users may not be expressively concerned with that part of the legislation.

Many participants in this study reported experiencing an increase in the price of vaping products, less product choice, and added inconvenience. Factors in the success of using e-cigarettes to stay stopped from smoking are not limited to e-liquid nicotine strength, but also include having a satisfying functioning vaping set-up which is affordable and convenient.^31^ Therefore, although not demonstrated in this study of mostly exclusive e-cigarette users, it is possible that the TPD regulations may have had the unintended consequence of making it more difficult for some smokers to quit using an e-cigarette, and warrants further investigation. These potential barriers to switching may be further compounded by TPD warning labels which have been found to deter smokers from using e-cigarettes,^45^ although similar messages have been shown to have the potential to deter never smokers from trying e-cigarettes.^46,47^ Harm reduction messages comparing e-cigarettes to tobacco, such as ‘Use of this product is much less harmful than smoking’,^48^ have been explored in relation to *e-cigarette* packaging and advertising,^45,48,49^ but future research could focus on their inclusion on *tobacco* packaging as suggested by participants in this study as a way of nudging smokers to switch to less harmful vaping.

Paradoxically, the TPD restrictions prompted some vapers to buy much higher concentrates and amounts of nicotine than they would have otherwise, through stocking up on large quantities of nicotine before the TPD came into force or purchasing nicotine shots to add to larger bottles of 0 mg/ml liquid. This behaviour, also noted elsewhere,^27,28^ contradicts one of the main objectives of the legislation, potentially posing greater safety risks, for example, accidental poisoning by swallowing. In addition, the TPD restrictions had prompted some participants to buy unregulated illegal products. Black market products may pose risks to consumers^35^ and safety was a significant worry for participants who perceived foreign vaping products to be inferior and more hazardous than EU produced products. Although likely to be used by only a minority of e-cigarette users in the United Kingdom, these results indicate that there is a black market offering products which are no longer legally available, such as nicotine strengths above 20 ml/mg, eliquid bottles larger than 10 ml, and prohibited components.

### Limitations

Although the sample can be considered large for a qualitative study, these findings may not be generalisable to the wider e-cigarette user population, and, therefore, while evidencing experiences of the TPD (in line with the study’s aim), they cannot give an indication of how widespread the issues discussed are. Indeed, the sample had disproportionate representation from white males, and the sample consisted mostly of consumers who were exclusive e-cigarette users who reported being impacted by the TPD. In contrast, it is likely that the vast majority of UK e-cigarette users will have not knowingly been affected by the changes. However, it is still important to listen to the views of those consumers that have been affected in order to improve future policy. For example, policy makers are unlikely to want anyone turning to the black market, and ways of limiting this should be considered. In addition, it is also important in reviewing policy to ask consumers what they value in legislation affecting them, irrespective of their awareness of current legislation; fortunately, despite over representation of some groups, the large sample enabled a range of perspectives to be reported, including women who were less likely to report awareness or impacts of the TPD. It would be helpful though to gain more views from minority groups, and those that had relapsed to smoking, to explore the full range of possible views and experiences of the regulations.

Another limitation was that the vast majority of respondents were from the United Kingdom; it is not clear whether the TPD was experienced similarly in other EU countries, although the same themes were identified in data from the small group of participants not residing in the United Kingdom. As expected, data generated via verbal interview were generally richer than data generated via the survey, although the same themes were identified through triangulation. It was beyond the scope of the project to obtain the views of smokers, never smokers, and young people, although it would be helpful to explore whether the TPD protects these groups as intended.

## Conclusion

This research indicates that awareness of the TPD was not universal and restrictions do not appear to have influenced participants’ smoking relapse behaviour. Consumers valued regulatory changes that supported informed decision making (eg, ingredients lists) and safety (eg, regulation of e-liquid contents). They responded negatively to changes that caused inconvenience and plastic waste (eg, smaller e-liquid refill bottles and tanks/cartridges). This research shows that the TPD legislation has prompted some consumers potentially to put their safety at risk by purchasing non-compliant products from the black market. The cost of these impacts needs to be balanced against the potential benefit of deterring non-smokers and children from vaping, and more research is needed to ascertain to what extent the legislation has achieved this benefit. The implications of our analysis suggest that, from a consumer perspective, future e-cigarette regulation should not further restrict liquid/tank volumes and nicotine concentration, but should focus on ensuring product safety, particularly around ingredients used in e-liquids. Public health bodies, Stop Smoking Services, and healthcare professionals should consider raising awareness of the regulations to smokers to offer reassurance about vaping products and e-liquid ingredients, for example, by signposting to educational materials.^50^ Vape retailers also have a responsibility to communicate to customers how aspects of the regulations are designed to protect consumers.

## Appendix 1

### Question phrasing

#### Survey

Do you live in an EU country (including the UK)?

○ Yes
○ No

*Display This Question*:

If Do you live in an EU country (including the UK)? = Yes

The Tobacco Products Directive (TPD) is an EU Directive which restricts liquid bottle sizes to 10 ml, tank sizes to 2 ml, and nicotine strength to 20 mg. Liquid has to have its ingredients listed on the label, and vaping products including hardware have to have a warning label on them stating they contain nicotine. How has the legislation affected you? (advantages/disadvantages, change in price, availability or effectiveness, changed you vaping behaviour? E.g. started home mixing, buy on the black market). Please comment in the box below:

___________________________________________

___________________________________________

___________________________________________

___________________________________________

___________________________________________

#### Interview

‘Last time we spoke to you was just before the Tobacco Products Directive legislation came in last May. Are you aware of the legislation?’ Give brief description of legislation: ‘It restricted the bottle sizes you could buy to 10 ml, tank sizes to 2 ml, and nicotine strength to 20 mg. It meant that liquid had to have its ingredients listed on the label and that vaping products, including hardware, had to have a warning label on them stating they contained nicotine: ‘What are your thoughts on this legislation?’ ‘Advantages/disadvantages?’ ‘Has this legislation affected you at all?’ ‘How?’ ‘Have you noticed a change to the price, availability, or effectiveness of products?’ ‘Have you changed your purchasing behaviour as a result?’ (Prompt for home mixing, online purchasing, black or second hand market, modifying).
